# The Impact of Acute Kidney Injury on the Risk of Mortality and Health Care Utilization Among Patients Treated With Polymyxins for Severe Gram-Negative Infections

**DOI:** 10.1093/ofid/ofy191

**Published:** 2018-08-03

**Authors:** Sarah Baradaran, Douglas J Black, Katelyn R Keyloun, Ryan N Hansen, Patrick J Gillard, Beth Devine

**Affiliations:** 1 Global Health Economics and Outcomes Research, Allergan plc, Irvine, California; 2 Department of Pharmacy, University of Washington School of Pharmacy, Seattle, Washington

**Keywords:** acute kidney injury, health care resource utilization, mortality, polymyxins

## Abstract

**Background:**

With the rise of antibiotic resistance, polymyxin use has re-emerged but with a concern of renal toxicity. This study aims to assess mortality, length of stay, and total hospitalization cost associated with acute kidney injury (AKI) among recipients of intravenous (IV) sodium colistimethate (CMS) or IV polymyxin B (PMB).

**Methods:**

We conducted a retrospective database analysis using the Premier database from January 1, 2012, through September 30, 2015. Adults ≥18 years of age who were admitted for inpatient treatment with ≥3 consecutive days of CMS or PMB were included. Generalized linear models compared patients who developed AKI with those who did not. Models were adjusted for patient and clinical characteristics.

**Results:**

A total of 4886 patients were included; 4103 patients received CMS, and 783 received PMB. In the multivariable analyses, the presence of AKI was associated with higher in-hospital mortality in both the CMS cohort (adjusted odds ratio [aOR], 2.3; 95% confidence interval [CI], 1.9–2.7; *P* < .001) and the PMB cohort (aOR, 2.7; 95% CI, 1.8–4.2; *P* < .001). In both cohorts, patients who developed AKI experienced longer hospital stays (9.7 days and 11.6 days in the CMS and PMB cohorts, respectively; *P* < .001). The mean total hospitalization costs for patients who developed AKI were $47 820 higher (95% CI, $34 918–$60 722) in the CMS cohort and $35 244 higher (95% CI, $17 561–$52 928) in the PMB cohort.

**Conclusions:**

The clinical and economic burden of AKI in the context of polymyxin use is substantial. The use of effective antibiotics with limited toxicity should remain a priority.

In the United States, approximately 1.7 million individuals develop healthcare–associated infections, resulting in >95 000 deaths per year [1]. Antibiotic-resistant pathogens are the cause of 14% of all healthcare–associated infections in acute care hospitals. A 2011–2014 report from the Centers for Disease Control and Prevention evaluated healthcare–associated infections in US hospitals and found the following to be the most commonly seen multi-drug resistant pathogens (out of 408 151 reported pathogens): *Escherichia coli* (15.4%), *Klebsiella pneumoniae* and *Klebsiella oxytoca* (7.7% collectively), and *Pseudomonas aeruginosa* (7.3%) [2]. With resistance of gram-negative organisms on the rise, treatment options for these pathogens are limited. Polymyxins (sodium colistimethate [CMS] and polymyxin B [PMB]) have re-emerged since their use in the 1960s due to their strong antimicrobial activity against resistant gram-negative bacteria [3–5]. Although these agents are effective against resistant gram-negative bacteria, adverse events associated with polymyxin use include hypersensitivity reactions, nephrotoxicity, and neurotoxicity (such as vertigo, paresthesia, confusion, vision impairment) [6].

Drug-induced nephrotoxicity, specifically acute kidney injury (AKI), is the main safety concern associated with use of polymyxins. Clinically, AKI is defined as an abrupt (within 48 hours) increase in serum creatinine (SCr) of 0.5 mg/dL or a 50% increase above baseline for at least 2 repeated measurements [7, 8]. Clinical assessment of AKI is variable, and there is no gold standard for classifying AKI severity. Previous studies have estimated the cumulative incidence of AKI among patients receiving PMB and CMS to be between 12% and 48% [9–11]. These studies suggest variability in estimation of AKI due to the differences in patient selection, risk factors, and definitions of AKI. To date, there are no published studies that estimate the economic burden, in terms of length of stay (LOS) and total hospitalization cost of AKI, in the context of inpatient polymyxin use. However, previous studies of AKI in general (without associated polymyxin use) have established a clinical and economic impact. A study conducted by Chertow et al. [12] describes an association between an increase in SCr of ≥0.5 mg/dL with a 6.5-fold increase in the odds of mortality, a 3.5-day increase in LOS, and a $7499 marginal increase in total cost. The widely different incidence rates and the gap in the availability of economic estimates of polymyxin-associated AKI support the need for this analysis. We sought to assess mortality risk, inpatient length of stay, and total hospitalization costs associated with AKI among people who receive either intravenous (IV) CMS or IV PMB.

## METHODS

We performed a retrospective cohort study of patients admitted to the hospital between July 1, 2012, and August 31, 2015. We used the Premier Hospital Database, which contains information for about 50 million admissions from approximately 700 acute care hospitals, representing approximately 20% of inpatient discharges in the United States. The database includes teaching and nonteaching institutions, as well as urban and rural facilities. The database is fully de-identified and compliant with the Health Insurance Portability and Accountability Act of 1996 (HIPAA).

Patients ≥18 years of age and treated with 1 of the 2 polymyxins for ≥3 consecutive days were included. Patients were identified using billing charge codes for either intravenous CMS or PMB. Patients were excluded if there was evidence of an International Classification of Diseases-9-Clinical Modification (ICD-9-CM) diagnosis code for cystic fibrosis (277.x) during admission. In addition, any patient in either cohort who received an inhaled form of polymyxin was excluded from the study. Patients who used polymyxins 6 months before admission were excluded from the study. For those patients who had more than 1 qualifying hospital admission during the study period, only the first such admission was included in the analysis.

### Patient and Clinical Characteristics

We extracted patient characteristics including age, gender, race, Deyo version of the Charlson Comorbidity Index (CCI), previous hospitalizations (all-cause) at the same institution, admission at the same institution with diagnosis of AKI within the previous 6 months from the date of admission, and chronic kidney disease (CKD) on admission [13]. Chronic kidney disease was identified using the following ICD-9-CM codes: 582.x–583.x and 585.x–587.x. A recent study validated these ICD-9-CM codes for CKD with a positive predictive value of 98.4% for a diagnosis of chronic renal insufficiency compared with the gold standard estimated glomerular filtration rate (eGFR) <60 mL/min/1.73 m^2^ [14]. We also evaluated a number of clinical characteristics including average duration of polymyxin use, primary diagnosis for admission (by ICD-9-CM diagnosis code), use of other concurrent nephrotoxic drugs, dialysis or kidney transplant, type of infection, and microbiological culture information (causative organism and resistance). Use of other concurrent nephrotoxic drugs (foscarnet, vancomycin, amphotericin B, aminoglycosides, or platinum chemotherapy agents) with ≥2 consecutive days of use or contrast dye overlapping with polymyxin use was evaluated during the patient’s admission [15]. Patients who were undergoing dialysis during admission were identified by ICD-9-CM diagnoses and procedure codes (V39.95, V45.1, V56.0, V56.1, V56.9, 39.95, 54.98, and 55.69). The type of infection was classified first by the presence of a Medicare Severity Diagnosis Related Group (MS-DRG) major diagnostic category of infection (00018) and then the corresponding primary ICD-9-CM diagnosis code.

In patients with available microbiological laboratory data, these data were used to identify the most common causative organisms and the organisms’ resistance information. A positive culture for a gram-negative infection of *Pseudomonas aeruginosa, Acinetobacter baumannii, Klebsiella pneumoniae, Escherichia coli,* or *Proteus mirabilis* was defined as either resistant or highly resistant. Resistance patterns were stratified by the number of antibiotics to which the organism was resistant (0, 1, 2, or 3+ antibiotics).

### Exposure and Outcomes

AKI was defined as the presence of an ICD-9-CM diagnosis code for AKI documented during the index admission (584.x). This claims definition has been previously validated to identify AKI in the inpatient setting with a positive predictive value >80% vs eGFR <60 mL/min/1.73 m^2^ [16, 17].

We evaluated inpatient mortality, health care resource utilization, and total hospitalization costs for the index admission. Mortality was assessed from the patient’s discharge status of the hospital admission. Length of hospital stay was extracted from the inpatient admission summary. The total hospitalization cost was also extracted from the admission summary and includes the total cost to treat the patient during admission.

### Statistical Analysis

Patient and clinical characteristics measured as either continuous or categorical variables were reported. Bivariate statistics were used to compare patient and clinical characteristics and outcomes in patients with and without AKI. Generalized linear models were used for the mortality, length of hospital stay, and total cost of hospitalization. Multivariable adjustments were made for outcomes (eg, mortality was adjusted for age, gender, CCI, and dialysis during hospital admission; LOS was adjusted for age, gender, and CCI; cost was adjusted for age, gender, and CCI). All analyses were stratified and reported separately by type of polymyxin. Analyses were performed using STATA, version 14.1. A significance level of alpha = .05 was used. A sensitivity analysis was conducted in both the CMS and PMB cohorts, excluding patients with a 6-month history of AKI, dialysis, or CKD.

## RESULTS

**Figure 1.**
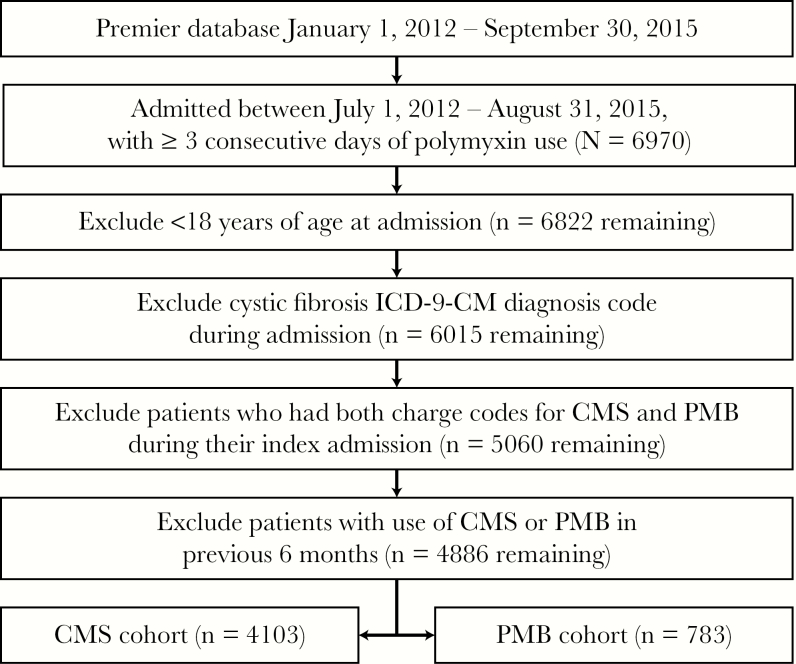
Patient selection flowchart. Abbreviations: CMS; sodium colistimethate; ICD-9-CM; International Classification of Diseases, Ninth Revision, Clinical Modification; PMB; polymyxin B.

We identified 6970 patients who were admitted between July 1, 2012, and August 31, 2015, who received at least 3 consecutive days of polymyxin therapy. After applying the inclusion and exclusion criteria, 4886 patients were included in our final analysis; 4103 (84%) patients received CMS at index admission (CMS cohort), and 783 (16%) received PMB at index admission (PMB cohort) (Figure 1). Microbiological culture data were available for 853/4886 (17%) of all patients, 744/4103 (18%) patients in the CMS cohort and 109/783 (14%) patients in the PMB cohort.

### 

#### Patient and Clinical Characteristics

In the CMS cohort, 2022 (49%) patients experienced AKI. The average age of the patients in the CMS cohort (SD) was 62 (16) years. Age was significantly different between patients who experienced AKI during admission and those who did not (64 years vs 59 years; *P* < .001). Patients who experienced AKI were also more likely to have a CCI of ≥3 (48% vs 29%, respectively; *P* < .001). Patients who experienced AKI had a significantly longer duration of CMS use compared with patients who did not experience AKI (8.1 days vs 7.6 days; *P* = .002) (Table 1; Supplementary Table 1).

**Table 1.** Patient and Clinical Characteristics

Patient Characteristics	CMS Cohort		PMB Cohort	
	With AKI (n = 2022)	Without AKI (n = 2081)	With AKI (n = 336)	Without AKI (n = 447)
Age, mean ± SD, y	64.0 ± 15.0^*^	59.4 ± 17.3	65.6 ± 14.4^*^	61.9 ± 17.0
Male sex, No. (%)	1194 (59)^*^	1147 (55)	186 (55)	228 (51)
Race, No. (%)				
White	1178 (58)	1268 (61)	196 (58)	253 (57)
Black	412 (20)^*^	369 (18)	71 (21)	74 (17)
Hispanic	175 (9)^*^	233 (11)	22 (7)	34 (7)
Other	427 (21)	432 (21)	69 (20)^*^	120 (27)
Unknown	5 (0.3)	12 (0.6)	0 (0)	0 (0)
Comorbidities during admission, Charlson Comorbidity Index score, No. (%)				
0	304 (15)^*^	575 (28)	52 (15)^*^	144 (32)
1	322 (16)^*^	503 (24)	46 (14)^*^	109 (24)
2	435 (22)	407 (20)	64 (19)	76 (17)
≥3	961 (48)^*^	596 (29)	174 (52)^*^	118 (26)
Previous 6-mo hospital visits (inpatient or outpatient), all cause, No. (%)	1155 (57)^*^	1294 (62)	183 (54)	241 (54)
Previous 6-mo hospital visits (inpatient or outpatient) with AKI, No. (%)	514 (25)^*^	286 (14)	86 (26)^*^	66 (15)
CKD during admission, No. (%)	341 (17)^*^	147 (7)	54 (16)^*^	27 (6)
Clinical characteristics	(n = 2022)	(n = 2081)	(n = 336)	(n = 447)
Average total duration of treatment (CMS or PMB), mean ± SD, d	8.1 ± 5.9^*^	7.6 ± 5.1	7.5 ± 5.5^*^	6.4 ± 4.1
Primary diagnosis during hospital admission^**a**^				
Septicemia, No. (%)	560 (28)^*^	496 (24)	82 (24)^*^	77 (17)
Septicemia, gram-negative organism, No. (%)	118 (6)^*^	77 (4)	22 (7)	16 (4)
Postoperative infection, No. (%)	NR	NR	10 (3)	13 (3)
Respiratory failure, acute and chronic, No. (%)	59 (3)^*^	87 (4)	8 (2)	9 (2)
Urinary tract infection, No. (%)	NR	NR	2 (0.6)^*^	14 (3)
Septicemia, *Pseudomonas*, No. (%)	65 (3)^*^	43 (2)	NR	NR
Pneumonia, *Pseudomonas*, No. (%)	33 (2)^*^	64 (3)	NR	NR
Use of other concurrent nephrotoxic drugs, No. (%)	584 (29)	612 (30)	124 (37)	153 (34)
Dialysis during admission, No. (%)^**b**^	478 (24)^*^	293 (14)	65 (19)^*^	41 (9)
Kidney transplant during admission, No. (%)	2 (0.1)	2 (0.1)	0 (0)	0 (0)
Transferred to the ICU during admission, No. (%)	1370 (68)^*^	1091 (52)	208 (62)^*^	169 (38)
Time spent in the ICU during admission, mean ± SD, d	14.9 ± 23.2^*^	8.8 ± 22.1	10.8 ± 16.5^*^	5.6 ± 14.4
Top 3 infection types, No. (%)^c^	(n = 972)	(n = 754)	(n = 155)	(n = 132)
Septicemia	922 (95)	720 (95)	142 (92)	118 (89)
Postoperative infection	30 (3)	18 (2)	10 (6)	13 (10)
Bacteremia	2 (0.2)	5 (0.7)	NR	NR
Causative organism, No. (%)^d^	(n = 379)	(n = 365)	(n = 48)	(n = 61)
*Pseudomonas aeruginosa*	166 (44)	183 (50)	27 (56)^*^	23 (38)
*Acinetobacter baumannii*	149 (39)	122 (33)	13 (27)^*^	10 (16)
*Klebsiella pneumoniae*	130 (34)^*^	100 (27)	11 (23)	11 (18)
*Escherichia coli*	64 (17)	61 (17)	4 (8)^*^	9 (15)
*Proteus mirabilis*	63 (17)	56 (15)	1 (2)^*^	5 (8)
Classification of organism, No. (%)	(n = 379)	(n = 365)	(n = 48)	(n = 61)
Resistant to 0 antibiotics	37 (10)	46 (13)	10 (21)	23 (38)
Resistant to 1 antibiotic	6 (2)	9 (2)	3 (6)	3 (5)
Resistant to 2 antibiotics	8 (2)	9 (2)	0 (0)	0 (0)
Resistant to ≥3 antibiotics	328 (87)	301 (82)	35 (73)	35 (57)

Abbreviations: AKI, acute kidney injury; CKD, chronic kidney disease; CMS, sodium colistimethate; ICU, intensive care unit; NR, not reported; PMB, polymyxin B.

^*^ *P* < .05.

^a^Limited to top 5 primary diagnoses during hospital admission.

^b^Patients who had received either intermittent or chronic dialysis, as coded by ICD-9-CM codes, could be included in the analysis, even if they did not meet criteria for having an AKI.

^c^Only 1726/4103 (42.1%) patients in the CMS cohort and 287/783 (36.7%) patients in the PMB cohort had a Medicare Severity Diagnosis Related Group major diagnostic category of infection (0018); top 3 infection types reported for CMS cohort; top 2 infection types reported for PMB cohort.

^d^Not mutually exclusive; patients can have more than 1 causative organism; only 744 patients in the CMS cohort and 109 in the PMB cohort had microbiological data.

In the PMB cohort, 336 (43%) patients experienced AKI. The average age of the patients in the PMB cohort (SD) was 63 (16) years. Age was significantly different between patients who experienced AKI during the index admission and those who did not (66 years vs 62 years; *P* < .001). Patients who experienced AKI were more likely to have a CCI of ≥3 (52% vs 26%, respectively; *P* < .001). Patients who experienced AKI had a significantly longer duration of PMB use compared with patients who did not experience AKI (7.5 days vs 6.4 days; *P* < .001) (Table 1; Supplementary Table 2).

### Outcomes

#### Mortality

In the CMS cohort, the presence of AKI during admission was associated with a 15% higher risk of mortality compared with those who did not experience AKI (95% confidence interval [CI], 10%–21%; *P* < .001). In multivariable regression, adjusted for covariates, there were significant differences in mortality (AKI vs non-AKI; odds ratio [OR], 2.3; 95% CI, 1.9–2.7; *P* < .001).

In the PMB cohort, the presence of AKI during the index admission was associated with a 17% higher risk of mortality compared with those who did not experience AKI (95% CI, 5%–30%; *P* < .05). In multivariable regression, adjusted for covariates, there were significant differences in mortality (AKI vs non-AKI; OR, 2.7; 95% CI, 1.8–4.2; *P* < .001).

#### Length of Stay

**Figure 2.**
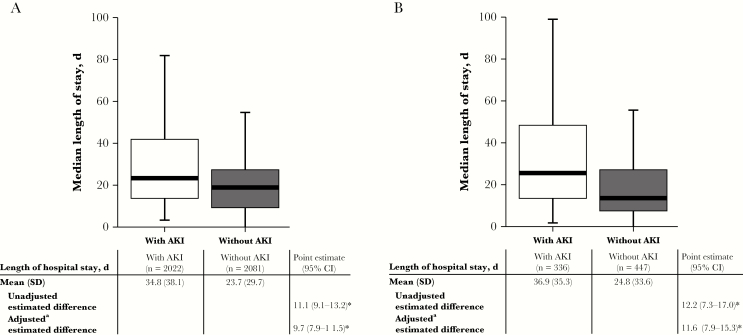
Health care utilization within the (A) CMS and (B) PMB cohorts. ^*^P < .001. ^a^Covariates: age, sex, CCI, previous AKI/CKD, number of days of CMS/PMB used. Abbreviations: AKI; acute kidney injury; CCI; Charlson Comorbidity Index; CKD; chronic kidney disease; CI, confidence interval; CMS; sodium colistimethate; PMB; polymyxin B.

In the CMS cohort, the presence of AKI was associated with an LOS 11.1 days longer (95% CI, 9.1–13.2) than in the absence of AKI. In multivariable regression, adjusted for covariates, there were significant differences in LOS (AKI vs non-AKI; estimated difference, 9.7 days longer; 95% CI, 7.9–11.5; *P* < .001) (Figure 2A).

In the PMB cohort, the presence of AKI was associated with an LOS 12.2 days longer (95% CI, 7.3–17.0) than in the absence of AKI. In multivariable regression, adjusted for covariates, there were significant differences in LOS (AKI vs non-AKI; estimated difference, 11.6 days longer; 95% CI, 7.9–15.3; *P* < .001) (Figure 2B).

#### Total Admission Costs

**Figure 3.**
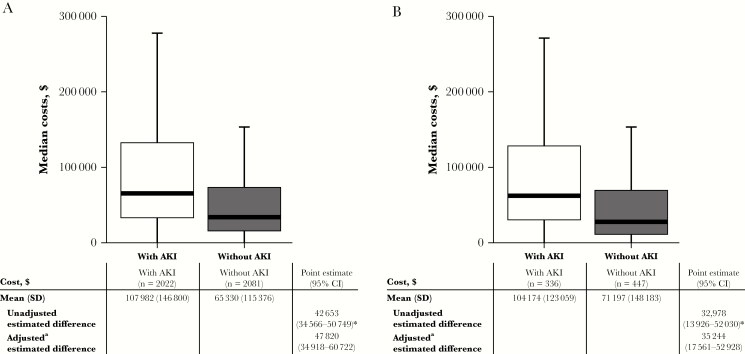
Total hospital costs for the (A) CMS and (B) PMB cohorts. ^*^P < .001. ^a^Covariates: age, sex, CCI, previous AKI/CKD. Abbreviations: AKI; acute kidney injury; CCI; Charlson Comorbidity Index; CI, confidence interval; CKD; chronic kidney disease; CMS; sodium colistimethate; PMB; polymyxin B.

In the CMS cohort, the mean total admission cost for patients who experienced AKI was $42 653 (95% CI, $34 566–$50 749) higher than the mean total admission cost of patients who did not experience AKI. In multivariable regression, adjusted for covariates, there were significant differences in mean total admission cost (AKI vs non-AKI; estimated difference, $47 820; 95% CI, $34 918–$60 722) (Figure 3A).

In the PMB cohort, patients who experienced AKI had a mean total admission cost $32 978 (95% CI, $13 926–$52 030) higher than the mean total admission cost of patients who did not experience AKI. In multivariable regression, adjusted for covariates, there were significant differences in mean total admission cost (AKI vs non-AKI; estimated difference, $35 244; 95% CI, $17 561–$52 928) (Figure 3B).

#### Sensitivity Analysis

In the CMS cohort, when all patients with a 6-month history of AKI, dialysis, or CKD were excluded, 2922 patients remained in the analysis. After adjusting for the same covariates as in the original models, the OR for risk of mortality in patients who experienced AKI compared with patients who did not experience AKI was 2.9 (95% CI, 2.4–3.5; *P* < .001). After adjusting for covariates, the hospital length of stay was 12.6 days longer in patients who experienced AKI compared with those who did not (95% CI, 10.4–14.8; *P* < .001). The adjusted mean total admission cost in patients with AKI was $58 666 (95% CI, $41 915–$75 417) higher than in non-AKI patients.

In the PMB cohort, when all patients with a 6-month history of AKI, dialysis, or CKD were excluded, 584 patients remained in the analysis. After adjusting for the same covariates as in the original models, the OR for risk of mortality in patients who experienced AKI compared with patients who did not experience AKI was 4.1 (95% CI, 2.5–6.8; *P* < .001). After adjusting for covariates, the hospital length of stay was 12.5 days longer in patients who experienced AKI compared with those who did not (95% CI, 7.5–17.6; *P* < .001). The adjusted mean total admission cost in patients with AKI was $38 897 (95% CI, $23 450–$54 344) higher than in non-AKI patients.

## DISCUSSION

AKI was associated with more than twice the risk of death during hospitalization. These patients experienced longer and 1.6 times more expensive hospital stays compared with those who did not have AKI. Furthermore, we found similar results after excluding patients with a possible history of kidney impairment. We also found that AKI is more common for patients who receive longer durations of polymyxin therapy.

Our findings support the established risk of AKI in the context of polymyxin use. Although previous studies have documented higher intensive care unit mortality in AKI patients, to our knowledge this is the first study to evaluate overall in-hospital mortality [18, 19]. Sekhri et al. [19] found no significant difference in length of hospital stay between AKI and non-AKI patients given polymyxins (*P* = .41), but because of the smaller sample size, the study may not have been adequately powered to detect a difference. To date, we have not found any other studies that have investigated the economic impact of AKI in the context of polymyxin use.

The limitations of this study are primarily based on using the Premier hospital database. The Premier hospital database lacks a date and time stamp associated with ICD-9-CM codes, which could have led to overestimation of AKI in this study. The database also prevents determination of previous hospital admissions at any hospitals other than the hospital of index admission due to the lack of a common identifier across hospitals, which also could have led to a biased sample of patients included in the AKI group. Third, CCI was calculated in this analysis using ICD-9 diagnoses codes, and an assumption was made that these chronic diseases, including CKD, were diagnosed before the use of polymyxins. Finally, this study was not intended to establish an association between the use of polymyxins and AKI, because there was no comparator group (eg, patients given an antibiotic other than polymyxin). The strengths of this study include a robust sample size that enables detailed characterization of in-hospital mortality, LOS, and total admission costs. Similarly, this is the first study to evaluate health care cost and LOS among those with AKI in a setting with CMS or PMB use.

In this retrospective cohort database analysis of patients who received polymyxin therapy, mortality, LOS, and total cost of hospitalization were significantly higher in patients who experienced AKI compared with those who did not. Our findings suggest that the clinical and economic burden of AKI among polymyxin recipients is substantial and that polymyxin therapy should be used judiciously. In the absence of a prospective study with integrated claims and electronic health record, our results support a critical evaluation of the risks vs benefits when considering polymyxin therapy.

## Supplementary Data

Supplementary materials are available at *Open Forum Infectious Diseases* online. Consisting of data provided by the authors to benefit the reader, the posted materials are not copyedited and are the sole responsibility of the authors, so questions or comments should be addressed to the corresponding author.

ofy191_suppl_supplementary_materials
