# Supplementary material for: The Impact of Acute Kidney Injury on the Risk of Mortality and Health Care Utilization Among Patients Treated With Polymyxins for Severe Gram-Negative Infections
Source: Open Forum Infect Dis. 2018 Aug 3;5(8):ofy191. doi: 10.1093/ofid/ofy191 (PMC6114194; doi:10.1093/ofid/ofy191)
Supplement: ofy191_suppl_supplementary_materials [file ofy191_suppl_supplementary_materials.pdf]

Supplemental Material

Supplementary Table 1. CMS Characteristics

Patient Characteristics	All patients (n=4103); n (%)	Patients with AKI (n=2022); n (%)	Patients without AKI (n=2081); n (%)	p-values[a]
**Age** (years) [mean ± SD]	61.7 ± 16.4	64.0 ± 15.0	59.4 ± 17.3	<0.001
**Gender** (male)	2341 (57%)	1194 (59%)	1147 (55%)	0.01
**Race**[b]				
White	2446 (60%)	1178 (58%)	1268 (61%)	0.08
Black	781 (19%)	412 (20%)	369 (18%)	0.03
Hispanic	408 (10%)	175 (9%)	233 (11%)	0.006
Other	859 (21%)	427 (21%)	432 (21%)	0.78
Unknown	17 (0.4%)	5 (0.3%)	12 (0.6%)	0.1
**Comorbidities during index admission** (Charlson Comorbidity Index [CCI])				
0	879 (21%)	304 (15%)	575 (28%)	<0.001
1	825 (20%)	322 (16%)	503 (24%)	<0.001
2	842 (21%)	435 (22%)	407 (20%)	0.12
3+	1557 (38%)	961 (48%)	596 (29%)	<0.001
**Previous 6-month hospital visits[c] (all-cause)**	2449 (60%)	1155 (57%)	1294 (62%)	0.001
**Previous 6-month hospital visits[c] with AKI**	800 (20%)	514 (25%)	286 (14%)	<0.001
**CKD during index admission**	488 (12%)	341 (17%)	147 (7%)	<0.001
**Clinical Characteristics**	**All patients (n=4103); n (%)**	**Patients with AKI (n=2022); n (%)**	**Patients without AKI (n=2081); n (%)**	**p-values[a]**
**Average total duration of CMS** (days)[mean ± SD]	7.8 ± 5.5	8.1 ± 5.9	7.6 ± 5.1	0.002
**Primary diagnosis for index admission**				
SEPTICEMIA	1056 (26%)	560 (28%)	496 (24%)	0.005
SEPTICEMIA, GRAM-NEGATIVE ORGANISM	195 (5%)	118 (6%)	77 (4%)	0.001
RESPIRATORY FAILURE, ACUTE & CHRONIC	146 (4%)	59 (3%)	87 (4%)	0.03
SEPTICEMIA—PSEUDOMONAS	108 (3%)	65 (3%)	43 (2%)	0.02
PNEUMONIA—PSEUDOMONAS	97 (2%)	33 (2%)	64 (3%)	0.002
**Use of other concurrent nephrotoxic drugs**	1196 (29%)	584 (29%)	612 (30%)	0.71
**Dialysis during index admission**	771 (19%)	478 (24%)	293 (14%)	<0.001
**Kidney transplant during index admission**	4 (0.1%)	2 (0.1%)	2 (0.1%)	1
**Transferred to the ICU during index admission n (%)**	2461 (60%)	1370 (68%)	1091 (52%)	<0.001
**Days spent in the ICU during index admission [mean ± SD]**	11.8 ± 22.8	14.9 ± 23.2	8.8 ± 22.1	<0.001
**Infection Type[d]**	**All patients (n=1726); n (%)**	**Patients with AKI (n=972); n (%)**	**Patients without AKI (n=754); n (%)**	**p-values[a]**
SEPTICEMIA	1642 (95%)	922 (95%)	720 (95%)	0.55
POSTOPERATIVE INFECTION	48 (3%)	30 (3%)	18 (2%)	0.38
BACTEREMIA	7 (0.4%)	2 (0.2%)	5 (0.7%)	0.15
**Causative Organism[e]**	**All patients (n=744); n (%)**	**Patients with AKI (n=379); n (%)**	**Patients without AKI (n=365); n (%)**	**p-values[a]**
Pseudomonas aeruginosa	349 (47%)	166 (44%)	183 (50%)	0.08
Acinetobacter baumannii	271 (36%)	149 (39%)	122 (33%)	0.10
Klebsiella pneumoniae	230 (31%)	130 (34%)	100 (27%)	0.04

Escherichia coli	125 (17%)	64 (17%)	61 (17%)	0.95
Proteus mirabilis	119 (16%)	63 (17%)	56 (15%)	0.63
**Classification of Organism**	**All patients (n=744); n (%)**	**Patients with AKI (n=379); n (%)**	**Patients without AKI (n=365); n (%)**	**p-values[a]**
Resistant to 0 antibiotics	101 (14%)	37 (10%)	46 (13%)	0.22
Resistant to 1 antibiotics	15 (2%)	6 (2%)	9 (2%)	0.39
Resistant to 2 antibiotics	17 (2%)	8 (2%)	9 (2%)	0.74
Resistant to 3+ antibiotics	629 (85%)	328 (87%)	301 (82%)	0.13

[a]p-values calculated from t-tests; [b]Not mutually exclusive; percentages may not add up to 100%; [c]Hospital visits include inpatient and outpatient visits at the same index hospital; [d]Only 1726/4103 (42.1%) of patients overall had a Medicare Severity Diagnosis Related Group (MS-DRG) major diagnostic category of infection (0018); [e]Not mutually exclusive; Patients can have more than one causative organism; Only 744 patients in the CMS cohort had microbiological data; *Note: Percentages do not add up to 100% due to conventional rounding.*

Supplementary Table 2. PMB Characteristics

**Patient Characteristics**	**All patients (n=783); n (%)**	**Patients with AKI (n=336); n (%)**	**Patients without AKI (n=447); n (%)**	**p-values[a]**
**Age** (years) [mean ± SD]	63.5 ± 16.1	65.6 ± 14.4	61.9 ± 17.0	0.001
**Gender** (male)	414 (53%)	186 (55%)	228 (51%)	0.23
**Race[b]**				
White	449 (57%)	196 (58%)	253 (57%)	0.63
Black	145 (19%)	71 (21%)	74 (17%)	0.1
Hispanic	56 (7%)	22 (7%)	34 (7%)	0.57
Other	189 (24%)	69 (20%)	120 (27%)	0.04
Unknown	0 (0%)	0 (0%)	0 (0%)	------
**Comorbidities during index admission** (Charlson Comorbidity Index [CCI])				
0	196 (25%)	52 (15%)	144 (32%)	<0.001
1	155 (20%)	46 (14%)	109 (24%)	<0.001
2	140 (18%)	64 (19%)	76 (17%)	0.46
3+	292 (37%)	174 (52%)	118 (26%)	<0.001
**Previous 6-month hospital visits[c] (all-cause)**	424 (54%)	183 (54%)	241 (54%)	0.88
**Previous 6-month hospital visits[c] with AKI**	152 (19%)	86 (26%)	66 (15%)	<0.001
**CKD during index admission**	81 (10%)	54 (16%)	27 (6%)	<0.001
**Clinical Characteristics**	**All patients (n=783); n (%)**	**Patients with AKI (n=336); n (%)**	**Patients without AKI (n=447); n (%)**	**p-values[a]**
**Average total duration of PMB** (days)[mean ± SD]	6.9 ± 4.8	7.5 ± 5.5	6.4 ± 4.1	0.001
**Primary diagnosis for index admission**				
SEPTICEMIA	159 (20%)	82 (24%)	77 (17%)	0.01
SEPTICEMIA, GRAM-NEGATIVE ORGANISM	38 (5%)	22 (7%)	16 (4%)	0.06
POSTOPERATIVE INFECTION	23 (3%)	10 (3%)	13 (3%)	0.95
RESPIRATORY FAILURE, ACUTE & CHRONIC	17 (2%)	8 (2%)	9 (2%)	0.73
URINARY TRACT INFECTION (UTI)	16 (2%)	2 (0.6%)	14 (3%)	0.01
**Use of other concurrent nephrotoxic drugs**	277 (35%)	124 (37%)	153 (34%)	0.44
**Dialysis during index admission**	106 (14%)	65 (19%)	41 (9%)	<0.001
**Kidney transplant during index admission**	0 (0%)	0 (0%)	0 (0%)	-------
**Transferred to the ICU during index admission n (%)**	377 (48%)	208 (62%)	169 (38%)	<0.001

Days spent in the ICU during index admission [mean ± SD]	7.8 ± 15.5	10.8 ± 16.5	5.6 ± 14.4	<0.001
**Infection Type[d]**	**All patients (n=287); n (%)**	**Patients with AKI (n=155); n (%)**	**Patients without AKI (n=132); n (%)**	**p-values[a]**
SEPTICEMIA	260 (91%)	142 (92%)	118 (89%)	0.52
POSTOPERATIVE INFECTION	23 (8%)	10 (6%)	13 (10%)	0.29
**Causative Organism[e]**	**All patients (n=109); n (%)**	**Patients with AKI (n=48); n (%)**	**Patients without AKI (n=61); n (%)**	**p-values[a]**
Pseudomonas aeruginosa	50 (46%)	27 (56%)	23 (38%)	0.05
Klebsiella pneumoniae	23 (21%)	13 (27%)	10 (16%)	0.17
Escherichia coli	22 (20%)	11 (23%)	11 (18%)	0.53
Acinetobacter baumannii	13 (12%)	4 (8%)	9 (15%)	0.30
Proteus mirabilis	6 (6%)	1 (2%)	5 (8%)	0.16
**Classification of Organism**	**All patients (n=109); n (%)**	**Patients with AKI (n=48); n (%)**	**Patients without AKI (n=61); n (%)**	**p-values[a]**
Resistant to 0 antibiotics	33 (30%)	10 (21%)	23 (38%)	0.06
Resistant to 1 antibiotics	6 (6%)	3 (6%)	3 (5%)	0.76
Resistant to 2 antibiotics	0 (0%)	0 (0%)	0 (0%)	-------
Resistant to 3+ antibiotics	70 (64%)	35 (73%)	35 (57%)	0.09

[a]p-values calculated from t-tests; [b]Not mutually exclusive; percentages may not add up to 100%; [c]Hospital visits include inpatient and outpatient visits at the same index hospital; [d]Only 287/783 (36.7%) of patients overall had a Medicare Severity Diagnosis Related Group (MS-DRG) major diagnostic category of infection (0018); [e]Not mutually exclusive; Patients can have more than one causative organism; Only 109 patients in the PMB cohort had microbiological data
